# “Major pathologic response” in lymph nodes: a modified nodal classification for non-small cell lung cancer patients treated with neoadjuvant immunochemotherapy

**DOI:** 10.1186/s40164-023-00401-6

**Published:** 2023-04-18

**Authors:** Hongsheng Deng, Shan Xiong, Ran Zhong, Yongmei Zheng, Hengrui Liang, Bo Cheng, Jianfu Li, Feng Li, Zhuxing Chen, Haixuan Wang, Jianxing He, Wenhua Liang

**Affiliations:** 1 Department of Thoracic Surgery and Oncology, State Key Laboratory of Respiratory Disease, National Clinical Research Center for Respiratory Disease, Guangzhou Institute of Respiratory Health, the First Affiliated Hospital of Guangzhou Medical University, No.151, Yanjiang Road, 510120 Guangzhou, China; 2 Department of Pathology, State Key Laboratory of Respiratory Disease, National Clinical Research Center for Respiratory Disease, Guangzhou Institute of Respiratory Health, the First Affiliated Hospital of Guangzhou Medical University, Guangzhou, China

**Keywords:** Non-small cell lung cancer, Neoadjuvant, Lymph node, Regression, Pathological response, Survival

## Abstract

We aim to examine the prognostic value of major pathologic response in metastatic lymph nodes (mLN-MPR) after immunochemotherapy in non-small cell lung cancer (NSCLC), and demonstrate the pathological characteristic of regression in mLN. Adult patients consecutively undergone neoadjuvant immunochemotherapy and radical-intent surgery for initial stage cIII NSCLC between 2020 and 2021 were included. Hematoxylin- and eosin-stained slides of paraffinembedded sections of the degree of pathologic response in the primary tumor (PT) and its paired involved LNs were reviewed. Imaging mass cytometry was conducted to quantify the immunological status. With 10% as residual viable tumor (RVT) cutoff, mLN-MPR (HR: 0.34, 95%CI: 0.14–0.78; P = 0.011, ref: mLN-MPR(-)) showed more significant correlation with DFS than ypN0 (HR: 0.40, 95%CI: 0.17–0.94; P = 0.036, ref: ypN1-N2). And mLN-MPR combined with PT-MPR, compared with ypN stage combined with PT-MPR (p-value: 0.030 vs. 0.117), can better distinguished the DFS curves of the 4 subgroups of patients. mLN-MPR(+)/PT-MPR(+) patients had the best prognosis compared with other subgroups. Pathologic responses of RVT in PT and paired regional LNs [MPR inconsistency rate: 21/53 (39.6%)], and across different LNs could be inconsistent, especially in squamous cell carcinoma. RVT% in mLNs after immunochemotherapy appeared to be polarized [16 (30.2%) cases with RVT ≥ 70%; 34 (64.2%) with RVT ≤ 10%]. Partial regression of LN metastasis could present with distinct immune subtypes: immune-inflamed or immune-evacuation subtype, and the former presented with higher CD3, CD8, and PD-1 expression in the invasive margin. mLN-MPR demonstrated a potential prognostic value in predicting DFS in patients treated with neoadjuvant immunochemotherapy, but further research is needed to validate its usefulness for other survival outcomes, including OS.

**Supplementary Information:**

The online version contains supplementary material available at 10.1186/s40164-023-00401-6.

To the editors

Neoadjuvant immunotherapy [1] has been actively employed in non-small cell lung cancer (NSCLC) treatment, which allows for broad immune activation of T-cell clones and could cause ‘nodal immune flare’ [2] (pathologic evidence of sarcoid-like granulomas) that possibly lead to superior lymph node (LN) downstage efficacy. Notably, the nodal downstaging efficacy of neoadjuvant chemoimmunotherapy for NSCLC is around 71.0% [3], while the rate for neoadjuvant chemotherapy only ranges from to 7.0% [4] to 32.5% [5]. Major pathological response (MPR) was known as a useful surrogate of neoadjuvant therapy response in a consensus statement by Hellmann et al. [6]. The definition intended MPR to refer to residual viable tumor (RVT%) ≤ 10%; however, most investigators use this terminology more liberally to refer to the response in the primary tumor (PT) rather than for LNs. Previously, Corsini et al. [7] have demonstrated that MPRypN0 represents the most favorable surrogate endpoint following neoadjuvant chemotherapy; however, this standard neglects the cases with partial regression in metastatic LNs (mLNs) that are prone to long-term survival. Thus, a more refined surrogate endpoint is in need. Herein, we hypothesized that MPR in metastatic LN [mLN-MPR(+)] was with favorable predictive efficacy for disease-free survival (DFS) in the neoadjuvant immunochemotherapy setting for NSCLC. This preliminary study also demonstrated the pathological characteristic of partial regression of LN metastasis.

Adult patients consecutively undergoing neoadjuvant immunochemotherapy between January 2020 to January 2021 and radical surgery for initial stage cIII NSCLC were included. In total, 53 patients were included. All cases had initial LN metastasis diseases, and most of the patients were with squamous cell carcinoma (LUSQ) [n = 35 (66.0%)] (Supplementary Table 1). Hematoxylin- and eosin-stained slides of paraffinembedded sections of the degree of pathologic response in the PT and its paired involved LNs were reviewed by two experienced pathologists. Calculation of RVT% was scored according to immune-related pathologic response criteria (irPRC) [8]. ypN0 was defined as pathologically no regional LN metastasis, PT-MPR(+) was defined as RVT ≤ 10% in PT, and mLN-MPR(+) was defined as mean RVT% ≤10% across mLNs specimen as recommend by Liu et al. [9]. After neoadjuvant immunochemotherapy, 31 (58.5%) of cases achieved PT-MPR(+), 34 (64.2%) achieved mLN-MPR(+), and 28 (52.8%) were classified as ypN0. The median postoperative follow-up time was 12.5 (95%CI: 9.6 to 15.0) months.

To determine the prognostic implications of mLN-MPR, univariable Cox model analyses for DFS were conducted (including factors of age, gender, histology, PT-MPR, mLN-RVT, mLN-MPR, mLN-MPR/PT-MPR response pattern, ypN stage, ypN stage/PT-MPR response pattern, and adjuvant treatment) (Table 1). Cumulative DFS stratified by mLN-RVT 0%, mLN-RVT 1–10%, and mLN-RVT 11–100% using a univariate cox proportional hazards model was shown in Fig. 1A, showing that mLN-RVT 1–10% have hazard ratio (HR) similar to that of mLN-RVT 0% (0.42 vs. 0.32). It revealed that PT-MPR(+) alone (HR: 0.43, 95%CI: 0.18–0.78; P = 0.184, ref: PT-MPR(-)) was associated with prolonged DFS. To be noted, mLN-MPR (+) (HR: 0.34, 95%CI: 0.14–0.78; P = 0.011, ref: mLN-MPR(-)) (Fig. 1B) showed a slightly more significant correlation with DFS than ypN0 (HR: 0.40, 95%CI: 0.17–0.94; P = 0.036, ref: ypN1-N2). And mLN-MPR combined with PT-MPR, compared with ypN stage combined with PT-MPR (p-value: 0.030 vs. 0.117) (Fig. 1C and D), can better distinguished the DFS curves of the 4 subgroups of patients(Fig. 1B and D). Patients who presented as mLN-MPR(+)/PT-MPR(+) (RVT ≤ 10% in PT and mLNs) had the best prognosis compared with other subgroups. These results indicate that the survival predictive value of mLN-MPR is independent of PT-MPR, and a cut-off value of 10% for mLN-MPR is reasonable.

**Table 1:** Univariable Cox model analyses for disease-free survival of patients with NSCLC receiving neoadjuvant immunochemotherapy

	Number of patients	Univariable	
		HR (95%CI)	P value
Age (continuous)	53	1.04 (0.98–1.10)	0.217
Gender			0.724
Male	47	ref.	
Female	6	1.26 (0.36–4.45)	
Histology subtype			0.984
LUSQ	35	ref.	
Non-LUSQ	18	0.99 (0.41–2.37)	
PT-MPR			0.047
PT-MPR(-)	22	ref.	
PT-MPR(+)	31	0.43 (0.18–0.99)	
mLN-RVT			0.039
mLN-RVT 11–100%	19	ref.	
mLN-RVT 0%	28	0.32 (0.13–0.78)	
mLN-RVT 1–10%	6	0.42 (0.11–1.61)	
mLN-MPR			0.011
mLN-MPR(-)	19	ref.	
mLN-MPR(+)	34	0.34 (0.14–0.78)	
mLN-MPR/PT-MPR response pattern			0.030
mLN-MPR(-)/PT-MPR(-)	10	ref.	
mLN-MPR(-)/PT-MPR(+)	9	0.45 (0.12–1.66)	
mLN-MPR(+)/PT-MPR(-)	12	0.35 (0.11–1.08)	
mLN-MPR(+)/PT-MPR(+)	22	0.21 (0.08–0.61)	
pN stage			0.036
N2-N1	25	ref.	
N0	28	0.40 (0.17–0.94)	
pN stage/PT-MPR response pattern			0.117
pN2-N1/PT-MPR(-)	13	ref.	
pN2-N1/PT-MPR(+)	12	0.73 (0.24–2.21)	
pN0/PT-MPR(-)	9	0.70 (0.22–2.28)	
pN0/PT-MPR(+)	19	0.24 (0.07–0.76)	
Adjuvant treatment			0.178
No	13	ref.	
Yes	40	0.55 (0.23–1.32)	

MPR, major pathologic response; mLN, metastatic lymph node; RVT, residual viable tumor; PT, primary tumor; LUSQ, lung squamous carcinoma

**Fig. 1:**
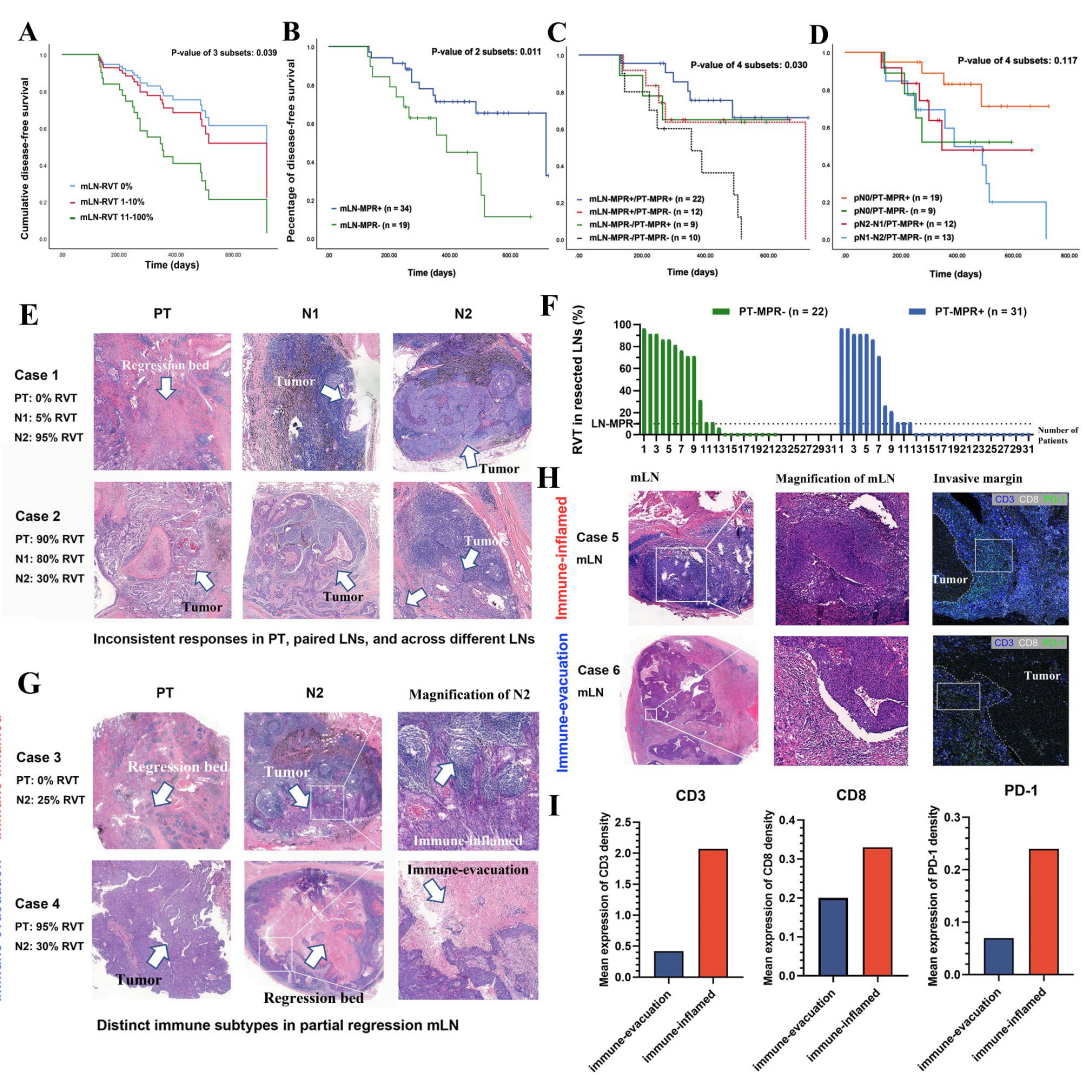
(A) Cumulative disease-free survival (DFS) stratified by mLN-RVT 0%, mLN-RVT 1–10%, and mLN-RVT 11–100% using a univariate cox proportional hazards model. (B) Kaplan-Meier survival curves of DFS stratified by (A) mLN-MPR(-) and mLN-MPR(+) subgroups (p value = 0.011); (C) stratified by mLN-MPR(+)/PT-MPR(+), mLN-MPR(+)/PT-MPR(-), mLN-MPR(-)/PT-MPR(+), and mLN-MPR(-)/PT-MPR(-) subgroups (p value = 0.030); (D) stratified by ypN0/PT-MPR(+), ypN0/PT-MPR(-), ypN1-N2/PT-MPR(+), and ypN1-N2/PT-MPR(-) subgroups (p value = 0.117) (E) Representative 2 cases of inconsistent pathologic responses of residual viable tumor in primary lesion and paired regional LNs, and across different LN stations (Case 1, Case 2). (F) Histogram showing the percent RVT in resected involved LNs of patients with PT-MPR(-) (left, green bar chart) and PT-MPR(+) (right, blue bar chart). Eight patients had PT-MPR(+) while RVT in mLN > 10%; and twelve patients had PT-MPR(-) but mLN reached mLN-MPR(+). (G) Typical examples of patients with partial regression of mLN exhibiting distinct immune-related phenotypes: immune-inflamed subtype (Case 3, LUSQ, with 0% RVT in PT, and 25% in N2 LN), and immune-evacuation subtype (Case 4, LUSQ, with 95% RVT in PT, and 30% in N2 LN). (H) Imaging mass cytometry image of invasion margin (stroma-tumor border area) from 2 partial regression mLN (Case 5, immune-inflamed subtype; Case 6, immune-evacuation subtype). The two solid line boxes represented the region of interests (ROIs). Blue color: CD3; White color: CD8; Green color: PD-1. (I) The histograms showing the CD3, CD8, and PD-1 densities, respectively, in ROIs between immune-inflamed subtype and immune-evacuation subtypes

Following a rigorous histopathological assessment procedure for slide handling, we found that the pathologic responses of RVT in PT, paired regional LNs, and across different LNs could be inconsistent (PT/mLN-MPR inconsistency rate: 39.6%). Nine out of thirty-one patients (29.0%) had PT-MPR(+) while RVT in mLN > 10%; and twelve out of twenty-two patients (54.5%) had PT-MPR(-) but reached mLN-MPR(+). Representative two cases of discordance pathological responses of RVT in PT and paired regional LNs, and across different LN stations were shown in Fig. 1E. Case 1 patient had 0% RVT in PT (PT-pCR(+)), 5% RVT in N1 station, while RVT in N2 station had little response to treatment (mLN-MPR(-)) (Fig. 1E, Case 1); Case 2 patient presented with 90% RVT in PT (PT-MPR(-)), 80% RVT in N1 station, while there remained only 30% RVT in N2 station (Fig. 1E, Case 2). In addition, RVT% in LNs after neoadjuvant immunochemotherapy appeared to be polarized [16 (30.2%) cases with RVT ≥ 70%; 34 (64.2%) with RVT ≤ 10%] (Fig. 1F), which was consistent with the article by Ling et al. [10] revealing that 42.9% of resected LNs had RVT ≤ 10%, while 57.1% of LNs had RVT% ranged from 60 to 90%.

We also found that partial regression (RVT > 10%, ≤ 50%) in mLN can present distinct pathological immune subtypes: immune-inflamed subtype (Fig. 1H, Case 3), or immune-evacuation subtype (Fig. 1H, Case 4). Case 3 patient had 0% RVT in PT (PT-pCR(+)) and 25% RVT in N2 LN and the N2 LN exhibited as the immune-inflamed subtype, with only a small cluster of tumor cells at the corner of the metastatic LN and were infiltrated by numerous lymphocytes. Case 4 patient had 95% RVT in PT and 30% RVT in LNM after immunochemotherapy; the N2 LN exhibited as the immune-evacuation subtype with large amounts of necrosis at the center and the remaining RVT around the metastatic LN. To quantify the cellular heterogeneity and immunological status at a protein level, we designed an imaging mass cytometry (IMC) [11] panel with 3 markers (CD3, CD8, PD-1) to image samples from 2 partial regression (RVT > 10%, ≤ 50%) mLN specimens presenting as immune-inflamed subtype (Fig. 1H, Case 5), and immune-evacuation subtype (Fig. 1H, Case 6). The densities of invasion margin CD3 (2.07 vs. 0.42), CD8 (0.33 vs. 0.20), PD-1 (0.24 vs. 0.07) were comparatively higher in immune-inflamed subtype than that of immune-evacuation subtype (Fig. 1I).

In the current ypTNM staging system [12], the ypN stage is categorized by the number and position of positive LN (ypN0-ypN3); whereas treated mLN with partial response is considered the same stage to mLN without any sign of regression. Therefore, ypN staging criteria may not provide an accurate assessment for mLN diseases. Herein, we proposed to utilize mLN-MPR (RVT% cutoff of 10%) instead of ypN0 as a more modified nodal staging criteria to be the surrogate endpoint for DFS in the neoadjuvant immunochemotherapy setting for NSCLC. This study focusing on the prognostic significance of mLN-MPR may shed some light on refining this criterion. Moreover, we found that: (1) neoadjuvant immunochemotherapy presented with superior LN downstage effect (52.8% cases staged as ypN0); (2) pathologic responses of RVT in PT and paired regional LNs, and across different LNs could be inconsistent; (3) RVT% in LNs after neoadjuvant immunochemotherapy appeared to be polarized; (4) partial regression (RVT > 10%, ≤ 50%) in mLN could present distinct pathological immune subtypes that is probably related to the prognosis. However, given the relatively small sample size of our study, further research is necessary to validate these findings. And investigations into the potential applicability of LN-MPR as a prognostic factor for other survival outcomes, such as OS, are warranted to comprehensively assess its clinical utility.

## Electronic supplementary material

Below is the link to the electronic supplementary material.

Supplementary Table S1. Demographic characteristics, clinical-pathological characteristics and survival outcomes of 53 study participants. 

## Data Availability

The datasets generated during and/or analyzed during the current study are available from the corresponding author on reasonable request.

## Abbreviations

NSCLC: Non-small cell lung cancer
LN: lymph node
MPR: major pathological response
RVT: residual viable tumor
PT: primary tumor
DFS: disease-free survival
mLN-MPR: major pathological response in metastatic lymph node
PT-MPR: major pathological response in primary tumor
imaging mass cytometry: IMC
