# Supplementary material for: “Major pathologic response” in lymph nodes: a modified nodal classification for non-small cell lung cancer patients treated with neoadjuvant immunochemotherapy
Source: Exp Hematol Oncol. 2023 Apr 18;12:40. doi: 10.1186/s40164-023-00401-6 (PMC10114379; doi:10.1186/s40164-023-00401-6)
Supplement: Supplementary file 1 — Supplementary Table S1. Demographic characteristics, clinical-pathological characteristics and survival outcomes of 53 study participants. [file 40164_2023_401_MOESM1_ESM.docx]

**Table 1.** **Clinical-pathological characteristics and oncological outcomes.**

| A | All patients (n=53) | |
| --- | --- | --- |
| **Age (Median, range)** | 57, 37-74 | |
| **Gender (Male/ Female)** | 47/6 | |
| **Histological type-no.** |  | |
| Lung squamous cell carcinoma | 35 (66.0%) | |
| Lung adenocarcinoma | 12 (22.6%) | |
| Lung lymphoepithelioma-like carcinoma | 5 (9.4%) | |
| Large cell lung cancer | 1 (1.9%) | |
| **Pre-treatment cTNM-no.** |  | |
| cT2-4N1M0 | 2 (3.8%) | |
| cT1-2N2M0 | 19 (35.9%) | |
| cT3-4N2M0 | 29 (54.7%) | |
| cT1-4N3M0 | 3 (5.7%) | |
| **Anti-PD-1 agents-no.** |  | |
| Sintilimab | 21 (39.6%) | |
| Nivolumab | 10 (18.9%) | |
| Pembrolizumab | 7 (13.2%) | |
| Camrelizumab | 8 (15.1%) | |
| Tislelizumab | 7 (13.2%) | |
| **Chemotherapy regimens with ICIs-no.** |  | |
| Paclitaxel^1^+Platin^2^ | 42 (79.2%) | |
| Pemetrexeddisodium+ Platin^2^ | 8 (15.1%) | |
| Gemcitabine+Platin^2^ | 3 (5.7%) | |
| **Treatment cycle (Median, range)** | 3 (2-8) | |
| **Clinical response-no.** |  | |
| Complete response | 1 (1.9%) | |
| Partial response | 30 (56.6%) | |
| Stable disease | 18 (34.0%) | |
| Progression disease | 0 | |
| Unevaluable | 4 (7.5%) | |
| B | All patients (n=53) | |
| **Pathological assessment of primary lesion-no.** |  | |
| Major pathologic response | 31 (58.5%) | |
| Complete pathologic response | 23 (43.4%) | |
| RVT > 10% | 22 (41.5%) | |
| **Pathological assessment of LNs-no.** |  | |
| RVT ≤ 10% | 34 (64.2%) | |
| mLN-pCR (ypN0) | 28 (52.8%) | |
| RVT > 10% | 19 (35.8%) | |
| **Postsurgical ypTNM^1^-no.** |  | |
| ypT1-4N2M0 | 11 (20.8%) | |
| ypT1-4N1M0 | 5 (9.4%) | |
| ypT1-4N0M0 | 14 (26.4%) | |
| ypT0N1-2M0 | 9 (16.9%) | |
| ypT0N0M0 | 14 (26.4%) | |
| C | All patients (n=53) |  |
| **Adjuvant treatment-no.** |  |  |
| Yes | 40 (75.5%) |  |
| Immune-chemotherapy | 29 (54.7%) |  |
| Immunotherapy | 3 (5.7%) |  |
| Chemotherapy | 3 (5.7%) |  |
| Radio-chemotherapy | 3 (5.7%) |  |
| Radio-chemotherapy + Immunotherapy | 2 (3.8%) |  |
| No | 13 (24.5%) |  |
| **Postoperative follow up (days, median, 95%CI)** | 381 (305-456) |  |
| **Recurrence/Progression rate at sensor-no.** | 18 (34.0%) |  |
| **Recurrence/Progression type-no.** |  |  |
| Bone metastasis | 3 (5.7%) |  |
| Bone and Lung metastasis | 2 (3.8%) |  |
| Brain metastasis | 2 (3.8%) |  |
| Lung metastasis | 6 (11.3%) |  |
| Lymph node metastasis | 5 (9.4%) |  |

^1^Paclitaxel chemotherapy included: Abraxane, Paclitaxel liposome, Docetaxel

^2^Plantin-based chemotherapy included: Carboplatin, Nedaplatin, Lobaplatin and Cisplatin.
